# Empirical Determination of Transitory and Persistent Delinquency in Chilean Youth: Validation of the Criminal Engagement Severity Scale “EGED”

**DOI:** 10.3390/ijerph19031396

**Published:** 2022-01-26

**Authors:** Sergio Chesta, Ricardo Pérez-Luco, Paula Alarcón, Lorena Wenger, Andrés Concha-Salgado, Eduardo García-Cueto

**Affiliations:** 1 Departamento de Psicología, Universidad de La Frontera, Temuco 4811322, Chile; sergio.chesta@ufrontera.cl (S.C.); paula.alarcon@ufrontera.cl (P.A.); lorena.wenger@ufrontera.cl (L.W.); andres.concha@ufrontera.cl (A.C.-S.); 2 Departamento de Psicología, Universidad de Oviedo, 33003 Oviedo, Spain; cueto@uniovi.es

**Keywords:** youth offender, transitory, persistent delinquency, validation, EGED

## Abstract

Evidence from several longitudinal studies has established the relevance of the approach of evolutionary criminology to understanding and intervening with adolescent criminal offenders, seeking to halt the criminal behavior before its potential consolidation in adulthood. The aim of this study is to present the psychometric properties of the Criminal Engagement Severity Scale (EGED) to discriminate between transitory and persistent delinquency in Chilean adolescents of both sexes. The characteristics of the sample are revealed through descriptive analyses, and evidence of validity and reliability is provided that show its discriminant capacity using ROC curves and odds ratios, measures of internal consistency (Cronbach’s α and McDonald’s Ω), intraclass correlation, and unidimensional statistics. The results indicate that the EGED adequately discriminates between transitory and persistent delinquency, so that its use in contexts of assessment and intervention with adolescent criminal offenders can be recommended, because it helps to determine the intensity of the intervention required.

## 1. Introduction

In recent decades, the assessment of criminogenic risks and needs has been an area of particular interest for professionals who intervene with offenders, making it an extended practice in prisons [1]. Such assessments gain particular importance in the case of adolescent offenders, given the impact that interventions have in that stage of development [2,3,4]. Juvenile delinquency tends to be among the key issues to address in different politico-institutional contexts, because the individual and collective needs of adolescent offenders must be addressed, as they are also a population with high indices of rights violations.

Although there are great differences among youth offender systems around the world, the general purpose is to control juvenile delinquency by reducing the risk of recidivism in the offender population and preventing its onset where there is no contra-normative behavior, protecting, in most cases, the fulfillment of child and adolescent rights.

Evolutionary criminology integrates the evidence of longitudinal studies, generating theories to explain the criminal career and its traits, expressed in a large corpus of literature that addresses the biopsychosocial, criminogenic risks that lead to its persistence, as well as the factors that affects its desistance. DeLisi and Piquero [5] identify four complementary theories (self-control, psychopathy, evolutionary taxonomy, and biosocial criminology) to understand criminal behavior as a construct derived from genetic, psychological, and socio-etiological forces working in unison; the relevance is that the four theories adopt a life cycle perspective, studying the evolutionary development of criminal activity.

Beyond the complementary theories, Marc Le Blanc [6,7] offers a more integrative theoretical development in the criminological context: the central object of study of evolutionary criminology is antisocial behavior in all its aspects; not just criminal behavior and its official records, but also its associated factors, and how it changes throughout the life cycle. The changes occur on the biological, psychological, interpersonal, social, societal, cultural, historical, and antisocial planes, which are interconnected, affecting several aspects of life. On this basis, a conceptual and methodological model is developed, that permits its theorization in evolutionary criminology, understanding the evolution of antisocial behavior, its hierarchical structure, descriptors of the antisocial career, its activation, worsening, and desistance, among others [8].

Le Blanc [7] proposes three metatrajectories (persistent, transitory, and common) that represent the full gamut of antisocial behavior, each with their own psycho-social traits. The persistent metatrajectory is characterized by greater intensity, violence, and severity in criminal and contra-normative behavior, with little prosocial engagement and greater antisocial modeling, low self-control, and ability to self-limit. The common metatrajectory (typical of the average adolescent) notes a more opportunistic, less intense, and less severe antisocial behavior, whereas the transitory one is between the persistent and the common.

The instrument to be described in this article, the Criminal Engagement Severity Scale (EGED), distinguishes between criminal types as understood in Moffitt’s evolutionary taxonomy [9,10]. In the same line of evidence as Le Blanc’s metatrajectories, Moffitt’s theory suggests that there are two groups of offenders with different etiological causes [11]. The first is a small, pathological population with observable antisocial tendencies in very early childhood that persist throughout most of the life cycle. This group is identified as persistent delinquency (PD), and lifelong criminal behavior may be the result of the interaction of neurological, genetic, and environmental risk factors [12,13] that translate into a series of generalized and prolonged behavioral problems. The percentage of adolescent offenders in Moffitt’s original study [9] was 5% of the sample; in Oceania and Europe it has been estimated that it ranges from 5 to 10%, and in the United States, studies vary from 0.5 to 7.4% [14]. Studies in countries with emerging economies, where the interventions with adolescent offenders are underdeveloped, the percentage seems to be greater: in Chile, 34.1% is reported in a region of the south [15], and in South Africa, it is 41.5% [16].

Different studies describe typologies differentiated within PD, being characterized, for example, according to low, medium, or moderate risk levels [17], or according to personality patterns [18,19,20], among others. In their meta-analysis, Jennings and Reingle [21] describe the range of trajectories in PD observed in the literature as being from two to seven groups, with the most frequent being three and four groups.

The second group of offenders presents an antisocial behavior limited to adolescence, or transitory delinquency (TD), which has its origins in social processes and begins in adolescence and desists in adulthood [9,13,19]. The young people in this group present antisocial behavior to reduce the anxiety they experience because of the maturity gap typical of adolescence. On reaching puberty, they are biologically mature, but socially they still have restrictions inherent to their age, whilst they are also increasingly responsible for their own actions. Contra-normative behavior is associated with adult behavior, thus reducing feelings of inadequacy [22]. As adolescence ends, the antisocial behaviors decrease, because the maturity gap is reduced and the contra-normative behavior is not needed to reaffirm adulthood. In addition, antisocial behavior becomes a hazard to reaching future goals and its rewards turn into costs, ending in its desistance [12]. The percentage of adolescence-limited offenders reported by Moore et al. [14] in Oceania and Europe is between 10 and 26%, and in the United States, studies vary from 4.6 to 11.6%. In Chile, Solís and Alvarado [15] report 61.9%, and in South Africa, 58.5% is noted [16]. It is worth noting that the measurements in the studies are taken with different criteria; for example, in some only those convicted are counted, and in others, those arrested and charged.

Although there is evidence of the difference between PD and TD, functional application of the differentiation criteria is problematic. Whitten, McGee, Homel, Farrington, and Ttofi [23] examined 38 functionalizations of PD and applied them to participants in the Cambridge Study in Delinquent Development. When applying these criteria, the average PD characterized a subject of low age at first offense, a longer criminal career with greater number and frequency of sentences; however, the cut-off points and criteria applied to each functionalization are highly variable, which leads to finding differences in the result of prevalence rates (from 1.24% to 29.53%), in the average age of onset (from 10.86 to 26.07 years), average duration of the criminal career (from 16.96 to 39.86 years), and average sentences (from 4.03 to 23.33), for example.

The distinction between adolescents who present TD and PD becomes relevant when defining intervention strategies; Bonta and Andrews’s risk principle [24] emphasizes the need for a precise and valid assessment of the recidivism risk to adjust the intensity of the intervention to the offender’s risk level: low for those at low risk (TD), and high for those at high risk (PD), where the impact of the intervention would be more effective at reducing the recidivism risk. This logic also requires the need for validated tools to assess the recidivism risk. The EGED, then, is an instrument that can distinguish between PD and TD and provides evidence for an initial assessment to orient decisions on the most appropriate services to render for each case.

The use of criminogenic risk assessment instruments has become widespread in many correctional institutions around the world [25,26], although only a few are adapted or created specifically to evaluate Latin American adolescent offenders [27,28,29,30]. The evidence indicates that risk assessments using structured methods and instruments result in more precise estimations than unstructured ones [2,31]. This impact is only observed when there has been adequate training in the use of the instrument, as well as a suitable institutional context for its implementation [26,32,33], as based on the risk–need–responsivity (RNR) model by Bonta and Andrews [24].

Several studies report that although the intervention programs have adopted the use of properly validated instruments, professionals do not necessarily design or perform interventions according the evidence they provide [26,33,34], or they are used for purposes other than those specified for the instrument [35]. In the study by Peterson-Badali et al. [36], the authors indicate that the needs identified in the assessment reports on adolescent offenders were not fully addressed by the program offering. In the case of education and employment needs, a maximum of 42% was reached, noting a minimum of 15% in antisocial attitudes. In four of seven types of needs considered, less than 25% were addressed, accounting for the disparity between evaluation and practice.

A balance must be maintained between needs and the most appropriate strategies to provide the service. The situation described in the previous paragraph occurs, partly, due to the poor training of the teams and little offerings in the network to provide specific services (e.g., drug treatment, mental health) appropriately, among other issues. However, it may also be due to how the interveners configure their strategies, favoring non-criminogenic focuses to obtain a more effective impact in the intervention, according to the RNR principle of specific responsivity [37].

Hamilton et al. [2] also recommend conducting psychometric studies on each population where use of the tool is required; often in the development of instruments these are assumed to be generalizable, but the diagnostic thresholds of the scores change between populations due to the differences between them and the changes in the local laws and provisions of specialized services, among others [32,38].

The aim of this study is to contribute evidence of the psychometric properties of the EGED to evaluate the general criminal risk levels in adolescents involved in criminal activities, understood as severity of criminal engagement. Evidence is also sought of the capacity of the instrument to discriminate between the two most significant forms of delinquency at this stage, TD and PD, thus contributing a simple tool to guide decision-making on the intensity of the interventions required in each case, within the framework of criminal penalty defined for the adolescent. This is consistent with the indications of Andrés-Pueyo and Echeburúa [39], Vincent, Guy, and Grisso [32], Haqanee et al. [37], Peterson-Badali et al. [36] and Nelson and Vincent [34].

## 2. Materials and Methods

### 2.1. Participants

A total of 537 adolescents, 478 boys and 59 girls, participated in the study, all convicted under the Adolescent Criminal Responsibility Act in Chile. A total of 29.1% were incarcerated, serving their sentence or in preventive custody at the time of the assessment, 45.6% were on probation, and 25.3% were in precautionary measure programs, or were given alternatives to incarceration or ancillary penalties for drug use. The average age was 16.8 years at the time of the assessment, and 66.6% committed their first crime before the age of 13. A total of ninety-eight percent have a family income below EUR 424 per month (minimum wage in Chile was EUR 306), 59.2% have completed 8 years of schooling (only elementary school in Chile, but equivalent to 2^nd^ year of first cycle of secondary school in Spain), and 35.5% are in treatment for drug use.

After categorizing the adolescents into those with and those without a record, and the exclusion of those who committed sexual crimes, the analyses were conducted on a total of 426 adolescents. A detailed description of this sample is given in Table 1.

The sampling was by convenience and in the context of a research project that involved the application of a battery of instruments that included the EGED. The adolescents come mainly from 4 regions in southern Chile (91.7%).

To calculate the inter-rater reliability, 5 experts in youth justice and 5 professionals with at least 5 years of experience working with offenders were asked to assess the items in three categories according to whether they considered them essential or not to differentiate between PD and TD. Characteristics of the judges are described in Table 2.

### 2.2. Instruments

#### 2.2.1. Criminal Engagement Severity Scale “EGED” (by its Spanish Acronym)

The EGED corresponds to an adaptation of the “Grille de gravité de l’engagement criminel” used in the Centre Jeunesse in Montreal, Canada [40]. It consists of 12 dichotomous items that are completed using structured professional judgment from what is observed in the young person’s life history; of these items, the first four are considered critical, because each by itself is indicative of PD. This version has changed from the original Canadian version to adjust it to the Chilean context. In 2010, a first analysis was carried out which showed adequate (but perfectible) levels of discrimination between PD and TD (using as a gold standard the professional qualification and evidence from other instruments developed in Chile). To improve the discrimination capacity of the instrument, it was decided to change the parameters of some items: the age for determining the early onset of delinquency was increased from 12 to 13 years old, and the number of offenses from 5 to 13. In addition, the four critical items are defined as those with the greatest discriminatory power when applying the gold standard, which is an innovation compared to the Canadian instrument.

The EGED evaluates maladjusted behaviors associated with breaking the law and specific features of the commission of crimes. The instrument’s items are:

1. Precocity;
2. Presence of criminal record;
3. Volume of crimes over 12;
4. Absence of tension during their actions;
5. Presence of crimes that violated a person’s integrity;
6. Presence of problematic alcohol or drug use, or both;
7. Use of weapons or tools in the commission of crimes;
8. Planned organization;
9. Presence of criminal accomplices;
10. Spends time with maladjusted peers or offenders;
11. Utilitarian motivation to commit crimes;
12. Destruction of objects during their actions.

The strength of the EGED is that it assesses the main static risks associated with persistent criminal behaviors in young people, determining an adolescent’s degree of criminal engagement, or adherence to a pattern of persistent criminal behavior. It synthesizes evidence that facilitates the decision or professional judgment on the type of delinquency an adolescent presents; when applying it, the emphasis is on the person who makes the assessment, who triangulates the data before scoring, consults two or more sources aside from the young person’s self-report, and considers the official criminal history and not the judicialized one. These sources of information can be people who have close knowledge of their life history and current situation (family, professionals from previous programs, teachers, partner, and friends, etc.), and documentation available about the young person (court files, information from previous interventions, previous records of sentences or measures, and official databases, etc.).

Given that the EGED is consistent with the RNR Model, it includes the principle of minimal risk; thus, it is suggested that when there is doubt in the assessment of an item, that it not be marked and the necessary investigation be performed to score with certainty, crossing several sources of information for completion only when this is clear, or agreed upon with the assessment team. Being an instrument of professional judgment, it is inadvisable to ask the young person the questions directly; this may prove misleading by not adequately contrasting the information. The items should be checked from the data collected (interviews and files), the observation of the young person’s behavior, and what light other applied instruments can shed at the same time. In this sense, it is recommended that more than one professional with knowledge of the case take part in its completion and, if feasible, that the work be conducted in a team, for example, in a case analysis meeting.

#### 2.2.2. Risks and Resources Evaluation Report (FER-R)

The Risks and Resources Evaluation Report (FER-R) has been in use in Chile since 2001, with its initial version being applied for nine years in programs for adolescent offenders on probation, until arriving at the revised version FER-R 3.0. It uses structured professional judgments to assess criminogenic risks and social adaptation resources. The risk indicators are grouped into two large categories: (a) behavioral risks detected by the system and (b) psycho-social risks, evaluating previous behavior, school risk, relationships with maladjusted peers, family risk, drug abuse, and negative attitudes or tendencies; in the adaptation resources, active personal resources are recorded, such as interests, cognitive and social competences, and family resources. In addition, it records the frequency and progression of crimes and information about previous interventions. The FER-R yields a maximum of 39 points for recidivism risk on its total scale and 18 points for resources, and each subscale has its own maximum score. Both the total scale and the subscales provide evidence to design an intervention plan with intensity and focal points differentiated for each case.

The FER-R has various psychometric studies with Chilean samples, conducted at different times [41,42]. It shows internal consistency reliability using Cronbach’s alpha, which fluctuates between 0.82 and 0.98 for its different subscales with a value from 0.94 for the total risk scale. The convergent validity was studied with the YLS/CMI-CL, obtaining a correlation of 0.914 for the total scores of the two instruments. To establish its predictive validity, a follow-up was performed on 101 adolescents, explaining 73% of the variance, with 68% success in the prediction of recidivism. Finally, the construct validity is obtained by a confirmatory bifactorial analysis, obtaining CFI = 0.965; TLI = 0.961, and RMSEA = 0.036 for the risk scale; thus, a good fit between the model and the data is confirmed.

#### 2.2.3. Self-Disclosed Delinquency Scale (EDA)

The Self-Disclosed Delinquency Scale (EDA) is a guided interview form based on the criminal categories formalized in the Adolescent Criminal Responsibility Act in Chile and operated by state institutions dealing with childhood [43]. Its objective is to record the history of crimes, judicialized or not, i.e., official and unofficial (hidden) delinquency, reported by the adolescent themself. It contains 67 items that ask about several types of crimes, organized into three sections: (a) burglaries and thefts, (b) assaults and abuses, and (c) other crimes. In each section, seven age ranges are asked about (≤8, 8–9, 10–12, 13–14, 15–16, 17–18, and ≥19).

The administration is via guided interview, asking the adolescent about the frequency with which they have enacted each behavior, to reconstruct the history of the crimes they recognize having committed; only the frequencies of self-reported crimes are recorded, for each age range, otherwise the check box stays blank. During the assessment, support is offered in answering questions, and specific experiences are asked about, taking advantage of the discussion about the evolutionary development of the criminal activity in the adolescent’s life, which deepens the dynamics, producing a fuller understanding of the case. The reliability analyses indicate adequate internal consistency for burglaries and thefts (0.95) and assaults and abuses (0.79), and moderate internal consistency for other crimes (0.60), due to the diversity of behaviors it records.

#### 2.2.4. Youth Level of Service and Case Management Inventory, Chilean Version (YLS/CMI-CL)

The Youth Level of Service and Case Management Inventory, Chilean Version YLS/CMI-CL is an adapted inventory of the French-Canadian version of the YLS/CMI, reviewed and complemented with a score guide [44] created for research purposes in collaboration with the Centre de Jeunes de Montréal. It is derived from the LSI-R, a tool created to assess criminogenic risk in adults in the US judicial system.

The YLS/CMI-CL has 42 items, divided into eight subscales: Previous and current offenses, family situation and parental role, education and employment, relationships with peers, substance abuse, free time use, personality and behavior, and attitudes and tendencies. Each item is encoded as present or absent according to the data collected by the evaluator and cross-checked by at least 3 different sources, giving a final score between 0 and 42 points. According to this total score, the adolescents are categorized into four recidivism risk levels: low, moderate, high, or very high, contributing to a broad and detailed view of the risk factors, specific needs, and receptivity of each young person. It has undergone validation studies in multiple countries, and in Chile it has shown good indicators for its use with young offenders [28], with a Cronbach’s alpha of 0.90 on the total scale and values from 0.57 to 0.85 on the subscales; the inter-rater agreement was calculated using Cohen’s Kappa coefficient (K_27_ = 0.514, *p* < 0.001) and the concurrent validity with the MACI scales, where the correlations ranged from 0.21 to 0.44, with the Tendency to Substance Abuse Scale and Transgressor Scale being the ones with the highest correlations. To give evidence of its discriminant capacity, the recidivist and non-recidivist groups are compared using an ROC curve, obtaining a large effect size (AUC = 0.82, *p* < 0.01).

## 3. Results

### 3.1. Ability of the EGED to Discriminate between TD and PD

To determine the critical items and their cut-off scores, the characteristics of the main predictors in the meta-analysis by Cottle et al. [45] for criminal recidivism were reviewed, these being precocity, volume of crimes, and the presence of criminal record; then the data were explored to determine the cut-off score and to look for other indicators that discriminate between transitory delinquency (TD) and persistent delinquency (PD).

A group of young people “with no record” was determined, where they scored zero on the scale “Previous and current offenses” on the YLS/CMI-CL and on the “Impact of previous interventions” on the FER-R; those that scored one or more on both indicators were categorized as “with a record”. With these criteria, a difference was established based on evidence between groups that have characteristics inherent to the TD and PD. This decision made it possible to fix the cut-off point for the criterion of previous criminal record, because the FER-R scale used for the criterion does not admit the commission of any crime for its record, and that of the YLS/CMI-CL tolerates up to three previous judicialized offenses. This leaves a tolerance limit of only one crime as evidence of TD, which is why the commission of two judicialized offenses gives evidence of being PD.

To determine the criterion of precocity, the age of criminal onset recorded in the FER-R was used for a total of 141 cases (5–18 years) of both sexes (M = 11.9; SD = 2.7), and it was analyzed by segmenting the base, where the group with a record (N = 62) obtained a mean M = 13.00 and median Mdn = 13.00; thus, it was established as a cut-off point to determine PD an age less than or equal to 13 years, meaning the commission of a crime before 13 years is evidence of PD.

To determine the criterion of volume of crimes, the total of self-disclosed crimes on the EDA (TTTD) was used, recorded for all the cases of both sexes in the sample (N = 537; M = 316.01; SD= 908.81), and an Mdn= 15.00 was reported on this variable in the group with no record. Because the variable presented an AS= 4.07, it was decided to use central robust estimators [46] as the localization statistic apart from the median, which are shown in Table 3.

Evaluating the evidence of the estimators, it was decided to leave the cut-off point at 12 for being the lowest value observed; i.e., it was considered that 13 or more crimes are indicative of PD.

Using the three previous criteria, the sample was distributed into two groups, including in the first group adolescents who presented (a) criminal beginning at 13 years or later; (b) maximum 12 self-disclosed crimes (as reported in the EDA); and (c) maximum one judicialization in addition to the current case, which would correspond to the TD group. Thus, they made up a group of 420 cases of PD and 107 cases of TD.

The scores of both groups were compared for the remaining nine items, and it was observed that “absence of tension” also appears as a predictor of static risk, because the cases of PD that had the item marked (n = 154, 36.7%) were proportionally much higher than in the other group (n = 8, 7.5%). The odds ratio of marking the item in PD compared to TD was 7.16, 95% CI [3.99, 15.13]; the decision to include it as a critical item was to support the professional judgment of PD, by accounting for the psychological dimension of criminal engagement.

Finally, with the scores on the continuous scale of the EGED (0–12 points) recorded for all the cases of both sexes in the sample (N = 537; M = 6.13; DS = 3.15), an ROC curve was calculated between the TD and PD groups, categorized according to professional judgment during the execution of the study, in which the data emerged, where the cases were described by the professional responsible under the supervision of the research team. The area under the curve was 0.97 (*p* < 0.01, 95% CI 0.94–0.99), which indicates a good discriminant capacity and a large effect size [47]. From the evidence of the analysis, it was decided to tolerate up to three points (sensitivity = 95.79, specificity = 81.82) on the scale as evidence of TD; it was determined so that four or more points on the EGED would be indicative of PD.

When applying the four criteria plus the cut-off, the sample was ultimately divided into 87 cases of TD and 440 of PD.

### 3.2. Evidence of Reliability

Internal consistency reliability of the instrument was adequate with α= 0.80 and Ω = 0.90, where it is considered that McDonald’s omega coefficient is more appropriate for the type of items on this scale [48]. We note in Table 4 that it was not necessary to eliminate any of the items if we evaluate the resulting coefficient according to the column “Cronbach’s alpha if the element has been suppressed”.

Consistency between judges was obtained using the intraclass correlation coefficient (ICC), which is an estimation of the reliability between evaluators, and that indicates what proportion of their grades represents an underlying construct; for this, a two-factor mixed model was used with absolute agreement, where the average measures were interpreted according to Landers [49]. The results are in Table 5.

The result indicates that there is 81.1% agreement in the evaluation of the underlying construct among the judges.

### 3.3. Evidence of Unidimensionality

Because the EGED is a unidimensional scale, we can give evidence of it using the indicators given by the program FACTOR 10.9.02 [50]; to achieve this, a parallel analysis based on a minimum rank factorial analysis (MRFA) is performed using a polychoric correlation matrix that estimates indices of proximity to unidimensionality [51].

The result of the parallel analysis suggests the retention of a single factor; the other indicators given correspond to that of Unidimensional Congruence (UniCo), the explained common variance (ECV) and the Mean of Item Residual Absolute Loadings (MIREAL), each with their corresponding value by item (I-UniCo, I-ECV, and I-REAL), described in Table 6. The value of the EGED for UniCo was 0.93, for ECV 0.87, and for MIREAL 0.20. This evidence reveals an essentially unidimensional structure.

### 3.4. Evidence of Validity by Item and Critical Items

Analyses of ROC curves for each item on the EGED were performed using the total score of the FER-R as a test variable; Table 7 presents AUC values with their respective confidence intervals.

With the four criteria plus the cut-off point, the ROC curve using the total score of the FER-R as a test variable yielded an under the curve of 0.91 (*p* < *0*.01, 95% CI 0.87–0.95), which implies a large effect size.

## 4. Discussion

The aim of this study was to evaluate the psychometric properties of the Criminal Engagement Severity Scale, EGED, in a sample of Chilean offenders, demonstrating its capacity to discriminate between two observable forms of delinquency in adolescence: transitory delinquency (TD) and persistent delinquency (PD).

Regarding reliability, both the α and Ω coefficients are found to be adequate for this type of scale and there is no need to eliminate any items [52,53]. The level reached by the intraclass correlation coefficient is graded as excellent according to Cicchetti’s criteria [54].

With respect to the evidence of unidimensionality, an essentially unidimensional structure is recognized [51], both by the results of the parallel analysis, and by the UniCo, ECV, and REAL values (at item and overall level). The item that presented the poorest fit was “planned organization”, which presented a low ECV and a high REAL; however, if the factorial saturations are reviewed, adequate values appear. In addition, the reliability levels are high with the different estimators.

For the evidence of validity by item, the areas under the curve show values that go from 0.70 to 0.88, which are satisfactory for such instruments [47,55].

Considering these results, from a strictly psychometric point of view, the EGED presents evidence that validates its use in assessment contexts to structure intervention plans for adolescent offenders according to the severity of their criminal engagement. Generally, in the specialized literature, such instruments are recommended, which is also reflected in the increase in their use in intervention programs with adolescent offenders [32,34]; although Latin America has instruments validated for populations in different countries [30], the effect of their use is diminished by the context in which the interventions must be implemented.

As for the criticisms previously mentioned in this article, regarding the need for training in the use of assessment instruments in a correctional context, the little correspondence between the needs assessed and the established intervention plans, and the use of non-validated instruments or with purposes other than that for which they are used, the EGED offers a robust predictive and discriminant power that constitutes evidence of support in professional decision-making in juvenile justice, in the design of intervention plans, and in the guidance for judicial measures and criminal sentences.

## 5. Conclusions

The evidence indicates that the instrument has appropriate psychometric properties for its use in Chile; therefore, the authors advise that the instrument continues to be studied to perfect and hone its use in other social and institutional contexts and with specific populations.

The purpose of the EGED is to determine the degree of criminal engagement to differentiate and adjust the intensity of the intervention properly. In an adolescent is more engaged in delinquency, the intervenor should differentiate that they had many opportunities to learn and reinforce previous (counter-normative) learning, and intervene using the strategies that best suit that young person.

In the case of TD, the aim is to reduce criminalization and contact with the justice system, thereby avoiding iatrogenic interventions, and favoring a rapid social reintegration in normalized contexts, but with the support required to reduce risk behavior and avoid recidivism and new arrests.

When distinguishing PD, the intensity of the intervention should be adjusted according to the offender’s involvement, following the principles of the RNR and their individual characteristics. This implies an adjustment in the frequency of intervention contacts in short periods of time, number of objectives to be addressed, variety of focuses, depth of the changes sought, and specificityof the support provided in the helping relationship; all this, regardless of the sanction determined by the court. In other words, the severity of the criminal engagement is directly associated to the intervention plans, not to the sanction; in no case should a relationship be established between EGED score and sanction, the relationship sought is between EGED score and intensity of the recommended intervention in cycles not exceeding 3 months.

In general, then, EGED seeks to contribute to differentiate between the characteristics of young people: the greater the criminal engagement, the greater the counter-normative learning, so interventions should be adjusted to deliver a service that gives more opportunities to the young person and decreases their criminogenic risk.

The limitations of this study and those of the instrument are similar to those for the other instruments used in the adolescent correctional context. The sample of women (10.56%) is very low, which may alter the validity results for this group; and the characteristics of the instrument have not been studied in an indigenous population in Chile either, representing 12.8% in the most recent census [56]. Its use in the case of sexual offenders is not advised, given that the content of the items does not discriminate in that population. Contra-normative behaviors in the context of the Internet, such as online scams, cyberstalking, and others, must also be explored with the tool, as it has been reported that they are relevant in the diagnosis of risks and needs [57].

## Figures and Tables

**Table 1:** Descriptive statistics for the analysis sample.

Variable	Categories	With a Record	With no Record
Sex	Male	207	174
	Female	14	31
Region	IX Araucanía	63	88
	X Los Lagos	41	32
	XIV Los Ríos	77	51
	RM Metropolitan RM	14	12
	Others	11	1
Crime	Crimes against property	179	157
	Crimes against persons	19	18
	Other offences	12	20
Type of program	Probation	116	20
	Imprisoned	66	119
	Precautionary measure	39	66
Attendance to drug program	No	92	154
	Yes	106	33
Ethnic origin *	Mapuche	25	34
	Not Mapuche	167	151
Average age		16,98	16,61
Average years of schooling		7,76	8,59

* The Mapuche are the Indigenous inhabitants of Chile and Argentina.

**Table 2:** Descriptive statistics for the analysis sample.

Judge	Profession	Characteristics
Judge 1	Lawyer	Master in Childhood, Adolescence, and Family Law, former director of SENAME *. Consultant on children’s rights, child protection system, juvenile justice, and family law for the Chilean government.
Judge 2	Criminologist	Ph.D. in Criminology and researcher at the Department of Criminology of the University of Cambridge. Acted as National Coordinator for Parole Sanctions in SENAME, participating in the design and implementation of the new policy for the Juvenile Justice System.
Judge 3	Psychologist	Chief Technician in a Parole Programme, formerly intervenor in the same programme (6 years).
Judge 4	Psychologist	Master in Public Policy, Economics, and Public Management. Head of the Department of Juvenile Justice at SENAME. Formerly head of the Department of Prevention and Social Reinsertion of the Ministry of Justice and developer of the Multisystemic Therapy Programme (MST) in Chile.
Judge 5	Psychologist	Senior Specialist in Citizen Security and Justice at the Inter-American Development Bank. Head of the Crime Prevention Unit of the Ministry of the Interior. In charge of the development and coordination of crime prevention projects, interpersonal violence, and social reintegration of criminal law offenders.
Judge 6	Psychologist	Master and Specialist in Legal and Forensic Psychology. Specialist in Intervention Models in Child and Adolescent Sexual Abuse. Postgraduate studies in Attachment and Parentality. Assessor of Socio-delictual Risk and delegate of parole in child and adolescent programs (4 years).
Judge 7	Sociologist	Research Area Coordinator of the Center for Studies on Citizen Security of the University of Chile. Specialist in Prevention of violence and crime in juveniles, and in the Prevention of Violence at the Early Education Level.
Judge 8	Social worker	Master in Family Studies and Development. Specialist in Gender Studies. Intervenor and supervisor of parole programs (6 years).
Judge 9	Social worker	Master’s degree in Psychology, specialist in psychosocial forensic intervention. Director, technical coordinator, and intervenor of Juvenile Justice teams (20 years).
Judge 10	Social worker	Coordinator, intervenor, and supervisor of programs for adolescent offenders, substance use, and mental health problems (14 years).

* SENAME is the Chilean National Service for Children and Youth.

**Table 3:** M estimators for the criterion of Volume of Crimes.

	Huber’s M- Estimator	Bi-Weight Tukey’s	Hampel’s M-Estimator	Wave Andrews
TTTD With No Record	18.28	12.27	15.73	12.20

**Table 4:** Cronbach’s alpha coefficient by item on the EGED.

Item	Scale Mean *	Scale Variance *	Corrected Total Correlation	Cronbach’s Alpha *
i01. Precocity	5.72	8.26	0.52	0.78
i02. Presence of criminal record	5.66	8.34	0.48	0.79
i03. More than 12 crimes committed	5.39	8.42	0.55	0.78
i04. Absence of tension during	5.83	8.52	0.47	0.79
i05. Presence of crimes that violated	5.44	8.76	0.37	0.80
i06. Presence of problematic	5.66	8.89	0.28	0.81
i07. Use of weapons or tools in the	5.69	8.35	0.48	0.79
i08. Planned organization	5.82	8.82	0.34	0.80
i09. Presence of criminal accomplices	5.4	8.47	0.52	0.78
i10. Spends time with maladjusted	5.36	8.75	0.43	0.79
i11. Utilitarian motivation to commit	5.63	8.23	0.52	0.78
i12. Destruction of objects during	5.89	8.65	0.45	0.79

* Measures estimated if the element has been suppressed.

**Table 5:** Intraclass correlation coefficient.

	Intraclass Correlation	95% Confidence Interval			F-Test with True Value 0		
		Lower Limit	Upper Limit	Value	gl1	gl2	Sig.
Average measures	0.811	0.59	0.94	6.51	9.00	99.00	0.00

**Table 6:** Evidence of Unidimensionality on the EGED.

**Item**	**I-UniCo**	**I-ECV**	**I-REAL**
i01. Precocity	1.000	0.974	0.123
i02. Presence of criminal record	1.000	0.991	0.067
i03. More than 12 crimes committed	0.998	0.945	0.205
i04. Absence of tension during their actions	1.000	0.994	0.054
i05. Presence of crimes that violated a person’s…	0.806	0.577	0.492
i06. Presence of problematic drug or alcohol use, or both	0.783	0.557	0.338
i07. Use of weapons or tools in the commission of crimes	0.999	0.963	0.136
i08. Planned organization	0.631	0.449	0.607
i09. Presence of criminal accomplices	1.000	0.998	0.033
i10. Spends time with maladjusted peers or offenders	1.000	0.999	0.016
i11. Utilitarian motivation to commit crimes	0.994	0.902	0.240
i12. Destruction of objects during their actions	0.999	0.967	0.134

**Table 7:** Indices of area under the curve and confidence interval for the EGED.

Item	AUC	95% CI	
i01. Precocity	0.77	0.71	0.82
i02. Presence of criminal record	0.79	0.74	0.84
i03. More than 12 crimes committed	0.79	0.73	0.85
i04. Absence of tension during their actions	0.76	0.71	0.82
i05. Presence of crimes that violated a person’s integrity	0.72	0.66	0.78
i06. Presence of problematic drug or alcohol use, or both	0.71	0.65	0.76
i07. Use of weapons or tools in the commission of crimes	0.79	0.75	0.84
i08. Planned organization	0.70	0.65	0.76
i09. Presence of criminal accomplices	0.84	0.79	0.89
i10. Spends time with maladjusted peers or offenders	0.89	0.85	0.92
i11. Utilitarian motivation to commit crimes	0.81	0.76	0.85
i12. Destruction of objects during their actions	0.78	0.72	0.84

* All the *p* < 0.01.

## Data Availability

The data presented in this study are available on request from the corresponding author. The data are not publicly available due to privacy restrictions.
